# Calreticulin accelerates corneal wound closure and mitigates fibrosis: Potential therapeutic applications

**DOI:** 10.1111/jcmm.18027

**Published:** 2023-11-20

**Authors:** Sarita Mishra, Miguel A. Manzanares, Justin Prater, David Culp, Leslie I. Gold

**Affiliations:** ^1^ Department of Medicine, Division of Precision Medicine New York University School of Medicine Langone Health New York New York USA; ^2^ Powered Research, Research Triangle Park North Carolina New York USA; ^3^ Department of Pathology New York University School of Medicine Langone Health New York New York USA

**Keywords:** calreticulin, corneal epithelial cells, corneal injury, fibrosis, keratocytes, migration, proliferation, TGF‐β, tissue regeneration, α‐smooth muscle cell actin

## Abstract

The processes involved in regeneration of cutaneous compared to corneal tissues involve different intrinsic mechanisms. Importantly, cutaneous wounds involve healing by angiogenesis but vascularization of the cornea obscures vision. Previous studies showed that topically applied calreticulin (CALR) healed full‐thickness excisional animal wounds by a tissue regenerative process markedly enhancing repair without evoking angiogenesis. In the current study, the application of CALR in a rabbit corneal injury model: (1) accelerated full wound closure by 3 days (2) accelerated delayed healing caused by corticosteroids, routinely used to prevent post‐injury inflammation, by 6 days and (3) healed wounds without vascularization or fibrosis/hazing. In vitro, CALR stimulated proliferation of human corneal epithelial cells (CE) and corneal stromal cells (keratocytes) by 1.5‐fold and 1.4‐fold, respectively and induced migration of CE cells and keratocytes, by 72% and 85% compared to controls of 44% and 59%, respectively. As a marker of decreased fibrosis, CALR treated corneal wounds showed decreased immunostaining for α‐smooth muscle actin (α‐SMA) by keratocytes and following CALR treatment in vitro, decreased the levels of TGF‐β2 in human CE cells and α‐SMA in keratocytes. CALR has the potential to be a novel therapeutic both, to accelerate corneal healing from various injuries and in conjunction with corticosteroids.

## 1 INTRODUCTION

Outside of the endoplasmic reticulum (ER), in other cellular compartments and the extracellular space, the calcium‐binding ER chaperone, calreticulin, (CALR) has been steadily gaining discoveries of new roles in physiology and disease with a deeper understanding of how exogenous CALR functions.^1^, ^2^, ^3^, ^4^ We have previously shown that topically applied CALR in porcine and diabetic murine *(lepR−/−)* wound models enhanced the rate and quality of healing by a tissue regenerative process, hallmarked by epidermal appendage neogenesis and lack of scarring.^2^, ^5^, ^6^ In full‐thickness excisional murine wounds, neogenic pigmented black hair grew in the healed CALR‐treated wounds compared with scarring shown in buffer‐treated wounds. No other protein has shown tissue regenerative capability as part of the wound healing process and furthermore, the CALR‐treated wounds healed by abundant extracellular matrix (ECM) induction (granulation tissue formation) without apparent induction of angiogenesis.^4^, ^7^

The unique anatomy and function of the eye dictate a different healing process than the skin. Following injury, surgical procedures such as, photoreactive keratectomy (PRK) to improve vision and the presence of diseases including diabetic keratopathy, the cornea requires unique conditions for healing namely, lack of vascularization of vessels emanating from the limbus area and exquisitely fine regulation of the repair process such that the inflammation and ECM induction, required for reconstruction of the wound defect, does not terminate in fibrosis/scarring, referred to as hazing of the cornea.^8^, ^9^, ^10^ Both blood vessels in the cornea and fibrosis severely compromise vision and can lead to blindness.

**FIGURE 1:**
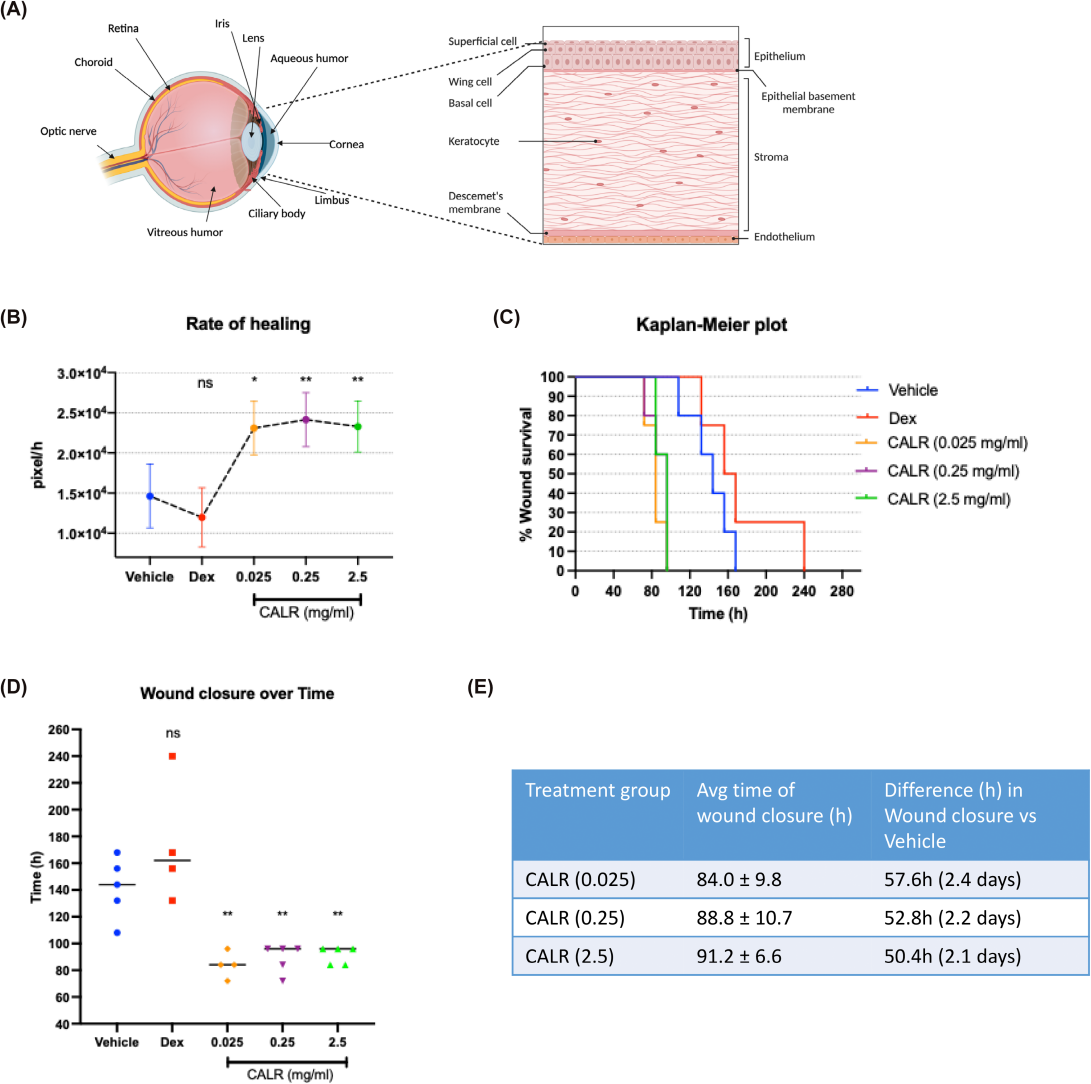
Calreticulin accelerates corneal wound closure in a rabbit partial keratectomy wound model. (A) Anatomy of eye; enlarged corneal region. Diagram was created using Biorender.com. The avascular cornea acts as a protective structural barrier and with the tear interface provides 2/3 refractive power of the eye.^8^, ^9^, ^10^, ^11^, ^12^ The lens refracts light and images for focus onto the retina. The iris regulates pupil size for the amount of light accessing the eye. The limbus forms the border of the cornea and opaque sclera and harbours limbal stem cells that replenish both the corneal epithelium and corneal stromal cells; the limbus is involved in blood vessel sprouting due to injury. The ciliary body aids in changing the shape of the lens. The aqueous humour is a nourishing clear fluid between the cornea and the lens that maintains ocular pressure. The vitreous humour maintains an oxygen gradient and shape of the eye. The choroid is vascular and provides oxygen and nutrients to the retina. The retina provides the neural signal to the optic nerve and the optic nerve transmits visual information to the brain. The cornea is composed of 4–5 layers of non‐keratinized stratified squamous epithelial cells (40–50 μm thick). The basal cell layer contacts the EBM (Bowman's layer) containing laminins, perlecan, nidogens and collagen I, III, V and VI. The stromal layer, comprising 90% of the corneal thickness contains and keratocytes that produce crystallins and collagens I, V, VI. Below the stromal layer is Descemet's membrane and a single layer of endothelial cells. Experiment #1: CALR dose ranging and efficacy in accelerating corneal wound closure. CALR (0.035 mL) was applied to the right non‐surgical and left eye injured by vacuum to the central corneal surface, BID for 14 days. Treatment groups: (1) negative control, vehicle, TBS; (2) 0.1% dexamethasone (anti‐inflammatory [Dex]); (3) CALR at 2.5 mg/mL [87.5 μg/application]; (4) CALR at 0.25 mg/mL [8.75 μg/application]; (5) CALR at 0.025 mg/mL [0.875 μg/application]; *n* = 5/group. Approximately 0.05 mL of 1% sodium fluorescein solution was applied to the ocular surface, followed by rinsing with phosphate buffered saline (PBS). Corneal staining was photographed under cobalt blue light using a Nikon Digital SLR camera on a tripod. The area in pixels of fluorescein staining at each time point was determined by Image J Software (NIH) of masked images. The fluorescein solution was applied two times per day (8–12 h apart), until Day 6 and once daily thereafter, and the area of corneal wound remaining open was measured as pixels [wound survival] (B) Rate of healing was measured by dividing the wound size on Day 0 by the wound size at the time of complete wound closure for each injured cornea. (C) Kaplan–Meier plot: the time to wound healing from 100% open at wound initiation is shown as per cent survival of the wound (y axis) over time (h; x axis) with the last animal to show full closure bisecting the x axis. CALR at 2.5, 0.25 and 0.025 mg/mL healed wounds by 96 h; TBS at 168 h, and Dex at 240 h. (D) Matrix Scatter Dot Plot: The time to wound closure for each injured rabbit cornea from each treatment group is shown by the Matrix Scatter Dot Plot. The mean values show accelerated wound closure in the three calreticulin treatment groups compared to TBS vehicle (*p* ≤ 0.01) and Dex (*p* ≤ 0.01). (E) The table provides the average hours to wound closure derived from the Matrix Dot Scatter plot, for the CALR treatment groups as compared to the TBS control. Data is represented as Mean ± SD; *n* = 5. [*p* ≤ 0.05 (*); *p* ≤ 0.01 (**)].

The cornea and the air‐tear film interface provide 2/3 of the refractive power of the eye.^11^ The outer layer of the cornea is composed of epithelial cells (CE cells) and functions to provide hydration and a barrier of protection against pathogens and injury (Figure 1A). The CE cells adhere to an acellular epithelial basement membrane (EBM) or Bowman's membrane composed of collagens I, III, V and VI.^9^ The stromal layer, composing 90% of the corneal thickness, lies below the EBM and contains sparse stromal cells, named keratocytes,^10^, ^12^, ^13^ that synthesize the stromal ECM composed of collagen fibers (I, V and VI) arranged as parallel lamellae that promote corneal transparency as well as conduits for cellular migration.^10^ Descemet's membrane mainly is produced by a single layer of endothelial cells lying directly below this membrane (Figure 1A).

Repair of the eye following injury or infection involves a delicate and finely balanced interaction between the CE cells and the underlying keratocytes. Injury to the cornea usually disrupts the EBM commencing a cascade of events.^8^, ^10^, ^14^ Specifically, IL‐1α and IL‐1β, constitutively expressed by the CE cells, in the absence of the intact EBM, have access to the keratocytes in the stroma, binds to their IL‐1 receptors causing Fas Ligand‐Fas receptor mediated apoptosis. Within 24 h, the remaining more distal keratocytes proliferate and become activated fibroblasts that release numerous cytokines and chemokines^8^ thereby attracting immune cells, including macrophages and fibrocytes from the bone marrow.^10^ TGF‐β2 and PDGF, released by CE cells directly cause activation/differentiation of fibroblasts and fibrocytes into myofibroblasts, that are opaque, express α‐smooth muscle actin (α‐SMA) stress fibres and produce a more disorganized scar‐type matrix compared to the transparent quiescent keratocyte.^9^, ^15^, ^16^, ^17^ Importantly, the fibrotic stromal wound healing response can be terminated and tipped towards regenerative healing by complete repair of the EBM and healthy keratocytes can eventually remove disordered matrix and clear corneal opacity.^16^ However, deeper corneal injury involving the endothelium and disruption of Descemet's membrane promote endothelial/stromal interactions leading to possible unresolved fibrosis and, the extension of injury deep into the limbal area (Figure 1A) induces blood vessel ingrowth to the center of the eye.^9^ Thus, the intensity and duration of the cytokine‐induced (e.g. Il‐1, Il‐6, TNF‐α) inflammatory response, which is dictated by the extent of injury and depth of the layers of the cornea impacted, albeit obligatory to healing, is commensurate with the extent of unresolved fibrotic hazing.^8^, ^9^

Prompted by our previous animal studies showing that topical CALR heals wounds without inducing angiogenesis, which is the critical requisite for healing ulcerations of the cornea, the current study was undertaken using a rabbit corneal injury model. The studies herein support the therapeutic use of CALR for the treatment of corneal injuries by accelerating wound closure and potentially mitigating the fibrotic response. Moreover, the data suggests that CALR can accelerate the delayed healing associated with standard of care (SOC) corticosteroid treatment.

## 2 MATERIALS AND METHODS

### 2.1 Materials

Recombinant human calreticulin (CALR) was synthesized in *Pichia Pastoris* (Intas Biopharma; Bangalore, India) and by tryptic digestion and sequencing was shown to be 85% pure (NYU Proteomics Core). CALR at 6.4 mg/mL was sterilized by 0.22 μm filtration, aliquoted and stored in 0.01 M Tris–HCl, 0.15 M NaCl (TBS), 0.003 M calcium, pH 7.5. Mitomycin C was from Sigma (#M5353) and 0.1% Dexamethasone sodium phosphate solution drops from Bausch and Lomb. Protein concentrations were determined using the BCA kit (ThermoScientific, Pierce, #23235). Epidermal Growth Factor was purchased from ThermoScientific (EGF, A42556) and Fibroblast Growth Factor from R&D Systems (FGF; #333FB010).

### 2.2 Rabbit corneal injury model and treatments

New Zealand White rabbits (*Oryctolagus cuniculus*), male (4–6 months old, 2.5–3.4 kg) were purchased from Covance (Indianapolis, ID) and acclimatized for 1 week. The studies followed The Guide for the Care and Use of Laboratory Animals reviewed and approved by IACUC at Powered Research, Triangle Park, NC (USDA approved). Prior to surgical partial keratectomy, animals received buprenorphine (0.01–0.05 mg/kg) subcutaneously and ketamine (50 mg/kg) and xylazine (10 mg/kg), intramuscularly. Baseline optical coherence tomography (OCT; Envisu, Leica) was performed followed by 5% betadine solution, ocular surface irrigation and application of the anaesthetic solution, 0.5% proparacaine and 10% phenylephrine HCL An 8 mm corneal vacuum trephine (Mori, Antony, France) was used on the left eye to create a central corneal defect through the epithelium into 10%–25% (~100 μm) of the stroma, the trephined section was removed creating the corneal injury, and antibiotic applied once.

#### 2.2.1 Experiment (Exp) #1

Dose‐range study: Five test treatments in 0.035 mL were applied to the surgical left eye (OS) and non‐surgical right eye (OD) at Day 0. Test treatments groups: (1) Vehicle, TBS; (2) Control, 0.1% Dexamethasone (Dex), a corticosteroid SOC used to control corneal inflammation; (3) 2.5 mg/mL CALR (87.5 μg); (4) 0.25 mg/mL CALR 8.57 μg (micrograms), (5). 0.025 mg/mL CALR (875 ng). *n* = 5 animals per treatment group. The test treatments were applied two times per day (BID) every 6–8 h for 14 days.

#### 2.2.2 Experiment (Exp) #2

CALR‐decreased Dex‐associated healing time. CALR in TBS was dialyzed against phosphate buffered saline (PBS). Six treatments in 0.035 mL were applied to OS surgical eye starting on Day 0. Treatment groups: (1) vehicle, PBS; (2) 0.0125 mg/mL CALR (438 ng); (3) 0.025 mg/mL CALR (875 ng); (4) 0.1% Dex; (5). 0.0125 mg/mL CALR (438 ng) plus 0.1% Dex; (6) 0.025 mg/mL CALR (875 ng) plus 0.1% Dex. *n* = 5 animals per treatment group. CALR was applied to OS eye one time per day (QD) for 4 days alone or in combination with 0.1% Dex, which was administered two times per day (BID) for 7 days or Dex alone. Eyes were harvested for histopathological analyses on Day 17 (Exp #1) and Day 28 (Exp #2). An intravenous overdose of Euthasol (100 mg/kg) was used for Euthanasia according to American Veterinary Medical Association (AVMA) guidelines.

### 2.3 Evaluations and analyses of corneas

Corneal baseline ocular examination (OE) and optical coherence tomography (OCT) were commenced prior to surgery on the left eye (OS). Immediately following surgery, the cornea was scanned by OCT, stained with 1% fluorescein sodium salt solution (Sigma, SKU #f6377), photographed, and treatments applied 15 min later. A. *Rate of wound closure*: Fluorescein ocular surface staining was photographed under cobalt blue light until the end of study at two times per day (8–12 h apart) and area in pixels determined by Image J. Kaplan–Meier plots for wound size survival (per cent survival) is shown at hours post‐injury. B. *OE scores*: assessment of corneal surface morphology and anterior chamber inflammation was quantified for OD and OS on post‐surgical Days 1, 3, 7, 14 and 17 [Exp #1] and on OS Day 28 [Exp #2] by the Hackett McDonald grading system^18^ using a Slit Lamp Biomicroscope (Kowa SL‐17 Portable Slit Lamp), and an indirect ophthalmoscope (Keeler 1205‐P‐1020 with a 28‐diopter Volk Lens). C. *OCT*: Anterior segment OCT quantifying depth of lesion, overall corneal thickness and stromal fibrosis was measured using callipers, contained in the OCT ophthalmascope *(*EnVisu, Leica) software, which was performed on post‐surgical Days 7 and 17 (Exp#1) and Day 7 and 28 (Exp#2).

### 2.4 Histopathology

Entire eyes including 1.0 cm of the optic nerve were harvested at the termination of Exp#1 and Exp #2, fixed in Davidson's solution overnight, transferred to 70% ethanol, cut, embedded in paraffin, sectioned 5.0 μm thick on slides, stained by haematoxylin and eosin and separately, by Masson trichrome, and the corneas scored for fibrosis and inflammation on a scale of 0–4.^19^

### 2.5 Immunohistochemistry

The protein levels of α‐SMA, vimentin were determined in the harvested rabbit eye tissue by immunohistochemistry using the Vectastain Elite kit (Vector laboratories, Burlingame, CA) essentially as described.^20^ The slides were scanned into the Omero Program by NYU Microscopy Core. Intensity of immunostaining of the injured cornea was determined using ImageJ.

### 2.6 Cell culture maintenance and treatments

Telomerase immortalized human CE cells (hTCEpi; CE/keratinocytes) from Danielle M Robertson (James Jester's laboratory) and primary human keratocytes were a kind gift from Dr. Shukti Chakravarty, Departments of Ophthalmology and Pathology, NYU School of Medicine. The CE cells were seeded and maintained in keratinocyte basal medium‐2 (KBM‐2) (#CC3103, Lonza BioScience) or containing KGM‐2 Bullets (Singlequote kit; #CC4152; Lonza BioScience) as keratinocyte complete growth media (KGM). Primary corneal keratocytes cells were maintained in Complete Media of DMEM/F12 (#11039021, Gibco) supplemented with 5% FBS, 1% Penicillin–Streptomycin solution (#30‐001‐CI, Corning) and 1 mM L‐ascorbic acid 2‐phosphate sesquimagnesium salt hydrate (#A8960, Sigma).

### 2.7 In vitro assay for cellular proliferation and migration

CE cells in KGM at a density of 1 × 10^4^ cells/well and corneal keratocytes in Complete Media at a density of 1 × 10^4^/well were separately seeded in 96 well plates. After 24 h, cells were synchronized in KBM‐2 basal media (CE cells) and DMEM‐F12 basal media containing 0.5% FBS (keratocytes) for 24 h followed by CALR treatment for 24 h. Metabolic activity, indicating number of cells, was determined by the CCK‐8 kit (#CK04‐05; Dojindo), and absorbance measured using a microplate reader (BioTek Synergy Neo2 Hybrid Multi‐Mode).

Cellular migration determined by the in vitro wound healing scratch plate assay. CE cells in KGM and keratocytes, seeded at 5 × 10^4^ cells/well in 24‐well plate were incubated in Complete Media until approximately 95% confluent (~24 h), treated with increasing concentrations of CALR and cell migration determined, as described.^6^ Cells were imaged by light microscopy at 0 h (Olympus CK2 microscope) and at the end of 8 and 16 h for CE and keratocytes, respectively; per cent wound closure was quantified by ImageJ.

Protein expression levels in corneal epithelial (CE) cells and keratocytes in response to calreticulin by Immunoblotting. CE cells were seeded at 2 × 10^5^ cells/well in KGM and keratocytes at 1.5 × 10^5^ in Complete Media in 6‐well plates and the cells cultured until 70%–80% confluency. The cells were treated with increasing concentrations of CALR in their respective basal media for 24 h. Cell lysates were prepared in cold RIPA buffer, protein concentrations determined by BCA kit (Pierce), followed by electrophoresis and immunoblotting for TGF‐β2, vimentin, and α‐SMA, and protein bands quantified by densitometry normalized to β‐actin or GAPDH, as loading controls.

### 2.8 Statistical analyses

The statistical significance between two different treatments (CALR concentrations, TBS and Dexamethasone) in the acceleration of wound closure compares the survival distribution of two samples (survival of the injury/wound) using the Log‐rank test (i.e. Mantel‐Cox test) as a nonparametric analyses of a test treatment measurement as the time/event of wound closure. Other in vivo and in vitro analyses used an unpaired two‐sided student's *t*‐test to estimate statistical significance between two groups (e.g. untreated control vs. treatment) at the 95% confidence level. All graphs were plotted using GraphPad Prism 9 software.

## 3 RESULTS

### 3.1 Calreticulin enhances the rate of corneal wound closure

The efficacy of CALR in tissue repair/regeneration of the rabbit cornea was determined in Exp #1. CALR over three logs concentration was topically applied to both eyes for 14 days and the tissue harvested after 17 days. Following the application of fluorescein to the ocular surface, the area of the wound was measured daily. CALR accelerated the rate of healing 1.6 times faster than the TBS vehicle control and two times faster than Dex, as determined by measuring the wound area (pixels) at Day 0 and daily thereafter divided by the hours(h) the wound reached full closure for each animal (Figure 1B). Represented by the Kaplan–Meier plot shown in Figure 1C, which illustrates the time to full closure from 100% open at initiation of the wound to the hour of complete wound closure (as % wound survival) of the last animal for each group (bisecting the x axis), all doses of CALR‐treated wounds were fully closed and healed by 96 h, which was 3 days faster than TBS‐treated wounds and 6 days faster than the Dex‐treated wounds. The Matrix Scatter Dot Plot in Figure 1D depicts the time to closure for each animal from each treatment group (bar = mean). Figure 1E shows the difference in days based on the average time to wound full closure of CALR versus TBS control. Without clinical reason, one animal each from the 0.025 mg/mL CALR and Dex‐treatment groups did not fully close by Day 17 post‐wounding; these animals were excluded from the data in Figure 1C,D. Table S1 shows the range and animal‐dependent variation for each treatment group; the animals without wound closure are highlighted. Notably, one rabbit each of the CALR at 0.25 and 0.025 mg/mL groups elicited eye wound closure as early as 72 h. Gleaned from this data, 100 times less CALR than the highest dose of 2.5 mg/mL was sufficient to achieve full closure of the wounds at an average of 91.2 h compared to Tris buffer vehicle at 141.6 h (*p* ≤ 0.01) and Dex at 174 h (*p* ≤ 0.01).

### 3.2 Calreticulin increases the rate of corneal healing delayed by dexamethasone

**FIGURE 2:**
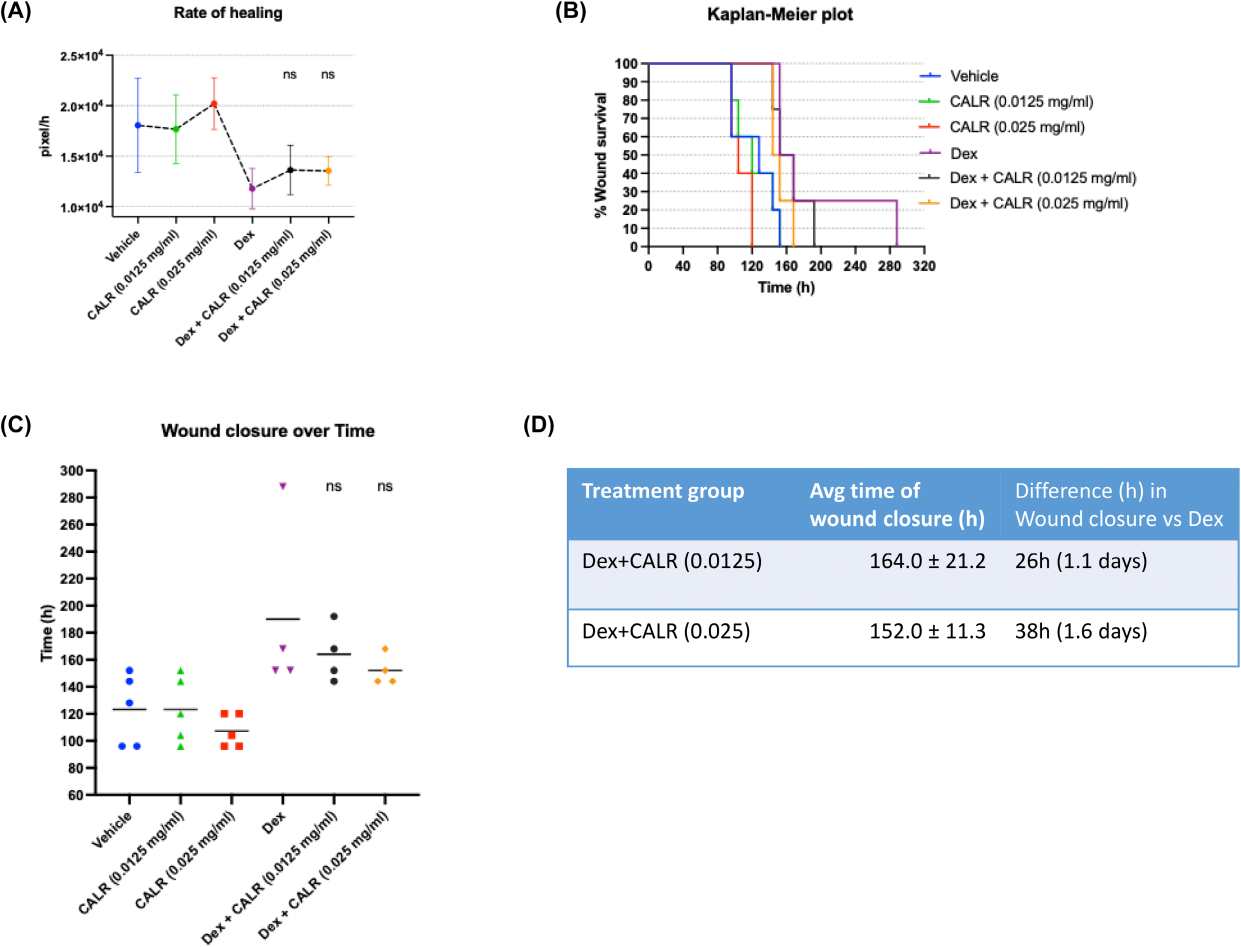
Calreticulin enhances wound closure of rabbit partial corneal keratectomies treated with Dexamethasone (Experiment #2). Rabbit left corneas were injured using the partial keratectomy vacuum model described in Methods and 0.035 mL CALR in PBS was applied to the left injured and right uninjured eyes one time per day for 4 days alone or with Dex applied twice per day for 7 days. Treatment groups: (1) negative control, vehicle PBS; (2) CALR at 0.0125 mg/mL (0.438 μg/application); (3) CALR at 0.025 mg/mL (0.875 μg/application); (4) 0.1% Dex; (5) Dex plus CALR at 0.0125 mg/mL (0.438 μg/application); (6) Dex plus CALR at 0.025 mg/mL (0.875 μg/application); (*n* = 5/treatment group). As described Figure 1, fluorescein solution was applied to the ocular surface before treatment, two times per day, until Day 6 and once daily thereafter, and the area of corneal wound remaining open was measured as mean pixel area (*n* = 6 animals) ± SD [wound survival]. (A) Rate of healing was measured by dividing the wound size on day 0 by the wound size at the time of complete wound closure for each injured cornea. (B) Kaplan–Meier Plot shows the time to wound healing from 100% open at wound initiation, shown as per cent survival of the wound (y axis) over time (h) with the last animal to show full closure bisecting the x axis: time of full closure: 0.025 mg/mL CALR at 120 h, PBS at 153 h, Dex at 288 h [range 152–288 h], 0.025 mg/mL CALR plus Dex at 168 h. (C) Matrix Scatter Dot plot represents the time of wound closure of each rabbit cornea from each treatment group. The mean values show time of wound closure (h) in the Dex plus CALR treated groups compared to Dex alone (not significant). (D) Calculated from the average healing value shown in Matrix Scatter Dot plot, the Table shows faster healing by CALR in combination with Dex compared to Dex‐treatment alone. One rabbit from each of three Dex‐treated groups was excluded due to lack of wound closure through 336 h post‐surgery. There was also an outlier in one DEX‐treated animal (healed at 288 h) skewing the average to be of slower healing. Data is represented as Mean ± SD; *n* = 5 [ns = not significant].

The potential use of CALR to obviate the notable delay in rate of healing caused by routine steroid treatment for inflammation of the eye has broad clinical translational potential.^9^, ^21^, ^22^ Based on the results from Exp#1, the lowest concentration, 0.025 CALR mg/mL and in addition, 0.0125 mg/mL were used in Exp #2 to improve the delay in healing caused by Dex. The graph in Figure 2A illustrates a small increase in the rate of healing with 0.025 mg/mL CALR treatment compared to the PBS control (*p* ≤ 0.393) and a similar small increase in rate of closure with 0.025 mg/mL CALR plus Dex compared to Dex alone (*p* ≤ 0.202). One animal per Dex‐treatment did not fully close through 28 days and therefore, the values were not included in Figure 2C showing average time to wound closure; highlighted in Table S2. Whereas PBS and 0.0125 mg/mL CALR both healed the corneal wounds at 123 h (5.1 days), 0.025 mg/mL CALR healed 0.6 days faster (107 h; 4.5 days) than PBS and 2.8 days faster than Dex alone (190 h; 7.9 days). The two groups receiving Dex in combination with CALR or Dex alone exhibited similar delays in healing. 0.025 mg/mL CALR plus Dex (152 h; 6.3 days) treated animals healed 1.6 days earlier than Dex alone (190 h; 7.9 days) (Figure 2D).

### 3.3 Calreticulin accelerates corneal wound closure with resolution of inflammation and fibrosis

**FIGURE 3:**
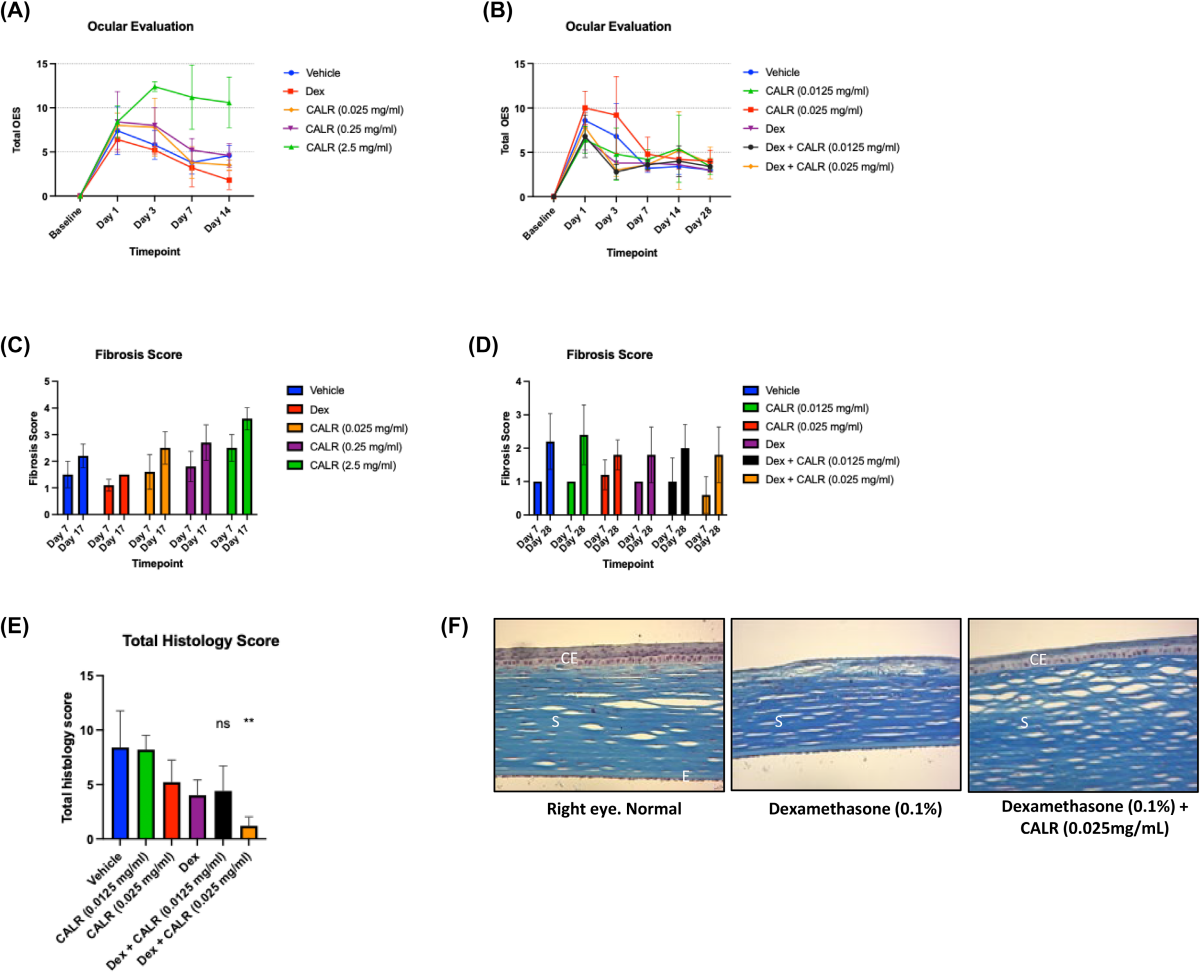
(A, B) Ocular Examination scores indicate that calreticulin treatment of rabbit injured corneas does not induce inflammation or fibrosis during healing. Corneal surface morphology and anterior inflammation of the cornea were evaluated using the Hackett McDonald grading system^18^ to obtain Ocular Examination (OE) scores for both the right uninjured and left surgical eyes as base line and on Days 1,3,7,14 and 17 (Exp#1) or Day 28 (Exp#2) using a Slit Lamp Biomicroscope and an indirect ophthalmoscope. Treatment groups are described in Figure 1. The highest score range for OEs is 34, which is weighted as follows, 10 attributable to the conjunctiva, 10 for the cornea, 13 for the iris, and 1 for the lens. (A) Exp #1: Total mean OE scores for the surgical eyes for each treatment group is shown over time (days). Mean OE scores for CALR 2.5 mg/mL treatment were higher from Days 3–17 than all other treatments whereas CALR 0.25 mg/mL and CALR 0.025 mg/mL treatments had mean OEs that that were similar to the vehicle control. (B) Exp #2: Treatment groups are described in Figure 2. Mean OE scores for the surgical eyes were highest on Day 1 post‐surgery for all treatment groups, which declined and showed comparable resolution of inflammation and surface irregularities for all six treatments from Day 7 to 28. (C, D) Anterior segment corneal Optical coherence tomography (OCT) indicates that calreticulin does increase corneal thickness or cause stromal fibrosis during healing. OCT was performed for all injured and corneas receiving treatments, as described in Exp 1 and Exp 2, to image the cornea for depth of lesion, overall corneal thickness and stromal fibrosis before and immediately after injury and then on Days 7 and 17 (Exp#1) and in addition, Day 28 (Experiment #2) post‐injury. Callipers, as part of the ophthalmoscope software, were used for measurements with scoring on a scale of 0 = Normal, 1 = Mild superficial area opacity, 2 = Moderate superficial corneal opacity, 3 = severe corneal opacity without full thickness involvement, 4 = Diffuse, sever, full thickness corneal opacity. (C) The bar graph shows OCT scores for fibrosis for Exp#1. Only CALR at 2.5 mg/mL gave a statistically significant score for fibrosis on Days 7 and 17 (*p* ≤ 0.0133, *p* ≤ 0.0009) post‐wounding compared to the Tris Vehicle control. (D) The bar graph shows that all treatment groups in Exp#2 gave similar fibrosis scores to the vehicle control and Dex scored on post‐surgical Days 7 and 28. (E) The bar graph shows the average for all the histology scores by staining with haematoxylin and eosin and Masson's Trichrome that are represented in Table 1 for each rabbit surgical eye in Exp#2. The compilation of the pathological scores for the 0.025 mg/mL CALR plus Dex treatment group were lower than the Dex alone group. PBS vehicle = 8.4 ± 3.4; 0.0125 mg/mL CALR = 8.2 ± 1.3; 0.025 mg/mL CALR = 5.2 ± 2.0; Dex = 4.0 ± 1.4; 0.0125 mg/mL CALR plus Dex = 4.4 ± 2.3; 0.025 mg/mL CALR plus Dex = 1.2 ± 0.8. (F) CALR 0.025 mg/mL plus Dex treatment of corneal injury shows similar histology to non‐surgical right eye after 28 days of healing from Exp#2. Images of corneal tissue stained for collagen density with Masson's Trichrome. Right non‐surgical eye normal eye, 0.1% Dex‐treated corneal injury, 0.025 mg/mL CALR plus 0.1% Dex treated injury. Data is represented as Mean ± SD; *n* = 5. [*p* ≤ 0.05 (*); *p* ≤ 0.01 (**)].

As shown in the graph in Figure 3A (Exp #1) with representative ocular surface morphology and inflammation scores (Ocular Evaluation (OE)/conjunctival redness) of both the right and left eyes, only the highest concentration of 2.5 mg/mL CALR gave OE scores that were higher compared to the lower doses of 0.25 and 0.025 mg/mL on Day 3 (12.4 vs. 8.0–7.8) through the end of the experiment, day17 (10.8 vs. 4.0). Notably, the right uninjured eye was unaffected by any treatments. OE scores for all group (Exp#2) treated with Dex alone or Dex plus CALR were similar on all days examined with complete resolution of inflammation of all treatment groups by Day 28 (3.0–4.0; Figure 3B). As the OE scores for CALR plus Dex treatment were no greater than Dex, used to control inflammation, the values reflect the normal healing process.

Regarding corneal thickness, as a measure of inflammation and stromal fibrosis, determined by anterior segment OCT, the data shows that treatment with 2.5 mg/mL CALR exceeded the original baseline mean thickness (459 ± 87 μm); all other treatment groups were smaller than each respective baseline at Day 17 (Exp #1; <300 μm; data not shown). For Exp #2, by Day 7, mean corneal thickness among all six treatment groups showed a similar decrease and by Day 28, corneal thickness was increased but not significantly (range: 278 ± 65–381 ± 72 μm). Fibrosis scores determined by OCT for Exp#1 showed that CALR treatment elicited a dose‐dependent increase in fibrosis measured on Days 7 and 17 post injury and that 0.025 mg/mL CALR was the same as TBS at Days 7 and 17 (Day 17 scores: CALR 2.5 mg/mL = 3.6, 0.25 mg/mL = 2.7, 0.025 mg/mL = 2.5, Tris = 2.2 [Figure 3C]). Thus, although there were apparent pathologies related to inflammation, the two lower concentrations of CALR with one log difference, barely induced a fibrotic response compared to TBS during healing over 17 days. Corneal fibrosis in Exp#2 (Figure 3D) was unremarkable on Days 7 and 28 for all groups with slightly less scores for all three Dex‐treatment groups on Day 28 post‐surgery.

Harvested eye tissues, stained with haematoxylin and eosin and Masson's Trichrome show high focal stromal fibrosis (score = 3) in the rabbit corneas treated with 2.5 mg/mL CALR compared with mainly mild fibrosis in all other treatment groups (Table 1). In addition, inflammatory infiltrate into the injured cornea for most animals was mild except for CALR (2.5 mg/mL), which was moderate (Score 2) in 4/5 animals (Exp#1). In Exp#2, inflammation was mild for most corneal tissues except for the PBS control (5/5 high). Surprisingly, CALR 0.025 mg/mL (3.5 μg total) plus Dex treatment scores varied from 0 to 1 and trended better than Dex‐treatment alone with respect to inflammation and fibrosis and far better than PBS or the 0.0125 mg/mL CALR lower concentration plus Dex. Specifically, CALR 0.025 mg/mL plus Dex group mainly scored 0 for fibrosis. Averaging all the histological categorical scores from Table 1 (Exp #2), as shown in Figure 3E, suggests that 0.025 mg/mL CALR might prevent fibrosis, particularly in combination with Dex. The images of the right uninjured cornea and the 0.025 mg/mL CALR plus Dex show comparable histology on the tissues harvested at Day 28 post‐surgery except the epithelial layer is thinner likely due to the Dex, which as shown has incomplete epithelial coverage (Figure 3F).

**TABLE 1:** Histologic evaluation of calreticulin‐treated rabbit corneal wounds.

Treatment Group	Rabbit #	CE and CS disorganization (0–4)	Inflammatory infiltrate CS (0–4)	Fibrosis stromal (0–4) haematoxylin and eosin	Fibrosis stromal (0–4) MT	Inflammation anterior uvea (0–4)	Inflammation posterior segment (0–4)
Vehicle	R1	1	0	1	1	0	0
	R2	1	0	1	1	0	0
Dex (0.1%)	R3	1	0	1	1	0	0
	R4	1	1	1	0	0	0
CALR (2.5 mg/mL)	R5	2	2	3	1	1	0
	R6	2	2	3	3	0	0
	R7	2	1	3	3	0	0
	R8	1	2	1	1	1	0
	R9	3	2	3	3	0	0
CALR (0.25 mg/mL)	R10	0	0	1	1	0	0
	R11	2	2	2	2	0	0
CALR (0.025 mg/mL)	R15	2	1	2	2	1	0
	R16	2	1	1	2	1	0
	R17	2	1	1	0	0	0
	R18	1	1	1	1	0	0
	R19	1	0	1	1	0	0

Treatment Group	Rabbit#	CE and CS disorganization (0–4)	Inflammatory infiltrate CS (0–4)	Fibrosis stromal (0–4) haematoxylin and eosin	Fibrosis stromal (0–4) MT	CL/C inflammation (0–4)	Inflammation anterior uvea (0–4)	Inflammation posterior segment (0–4)
Vehicle	R1	1	1	1	0	0	0	0
	R2	1	2	2	2	1	0	0
	R3	2	2	3	2	1	0	0
	R4	1	2	3	2	1	0	0
	R5	2	2	3	3	1	1	0
CALR (0.0125 mg/mL)	R6	2	1	3	2	1	0	0
	R7	1	1	3	2	1	0	0
	R8	1	1	2	2	0	0	0
	R9	2	2	2	2	1	0	0
	R10	2	2	2	2	1	0	0
CALR (0.025 mg/mL)	R11	1	2	1	1	0	0	0
	R12	1	1	1	2	0	0	0
	R13	0	1	1	0	0	0	0
	R14	2	2	2	1	0	0	0
	R15	2	1	2	1	1	0	0
Dex (0.1%)	R16	2	1	1	1	0	0	0
	R17	1	1	2	1	0	0	0
	R18	0	1	1	0	0	0	0
	R19	1	1	1	0	0	0	0
	R20	1	1	2	1	0	0	0
Dex (0.1%) + CALR (0.0125 mg/mL)	R21	0	0	1	1	0	0	0
	R22	0	1	1	1	0	0	0
	R23	1	1	2	1	0	0	0
	R24	2	2	2	1	1	0	0
	R25	1	1	1	1	0	0	0
Dex (0.1%) + CALR (0.025 mg/mL)	R26	0	0	0	0	1	0	0
	R27	0	0	0	0	0	0	0
	R28	0	0	1	0	0	0	0
	R29	0	0	1	1	0	0	0
	R30	0	1	1	0	0	0	0

*Note*: Following euthanasia, eyes including 1.0 cm of the optical nerve, were fixed in Davidson's solution overnight, transferred to 70% ethanol, cut, embedded in paraffin and sectioned parallel to the ciliary artery to include the cornea, anterior chamber, iris, lens, ciliary body, retina, vitreous chamber choroid and sclera and 5 μm thick serial sections placed to slides. Tissues from Day 17 and 28 post injury for Exp#1 and Exp #2, respectively, were stained with haematoxylin and eosin and scored by a grading scale modified from Tilton et al^19^ for severity of inflammation (0–4) by the following criteria: 0 = Normal tissue, 1 = Dilated iris vessels and thickened iris stroma with exudate, scattered inflammatory cells in anterior chamber, 2 = Inflammatory infiltrate into stroma of the iris, and/or ciliary body, moderate number of inflammatory cells in the anterior chamber, 3 = Heavy inflammatory infiltrate within iris stroma and ciliary body, heavy infiltrate in anterior chamber, 4 = Heavy exudate of cells and dense protein aggregation in anterior chamber and inflammatory cell deposits in corneal endothelium. In addition, the tissues on slides were stained for collagen with Masson's Trichrome to evaluate collagen density or deposition of collagenous material during wound healing and scar formation, by light microscopy. The Masson's Trichrome (MT) Scoring Scheme (0–4) was used to determine collagen density or deposition of collagenous material during wound healing and scar formation using the following criteria confined to the cornea: white = 0 = Normal, grey = 1 = mild inflammatory infiltration or mild fibrosis, blue = 2 = Moderate inflammatory infiltration and fibrosis, yellow = 3 = severe focal infiltrate and fibrosis, red = 4 = severe diffuse infiltrate and fibrosis. The histological categories evaluated were corneal epithelial and stromal disorganization, inflammatory infiltrate, stromal fibrosis (haematoxylin and eosin), stromal fibrosis (trichrome), corneal limbal and conjunctival inflammation, anterior segment inflammation and posterior segment inflammation.

### 3.4 Calreticulin stimulates proliferation and induces migration of CE cells and stromal keratocytes

**FIGURE 4:**
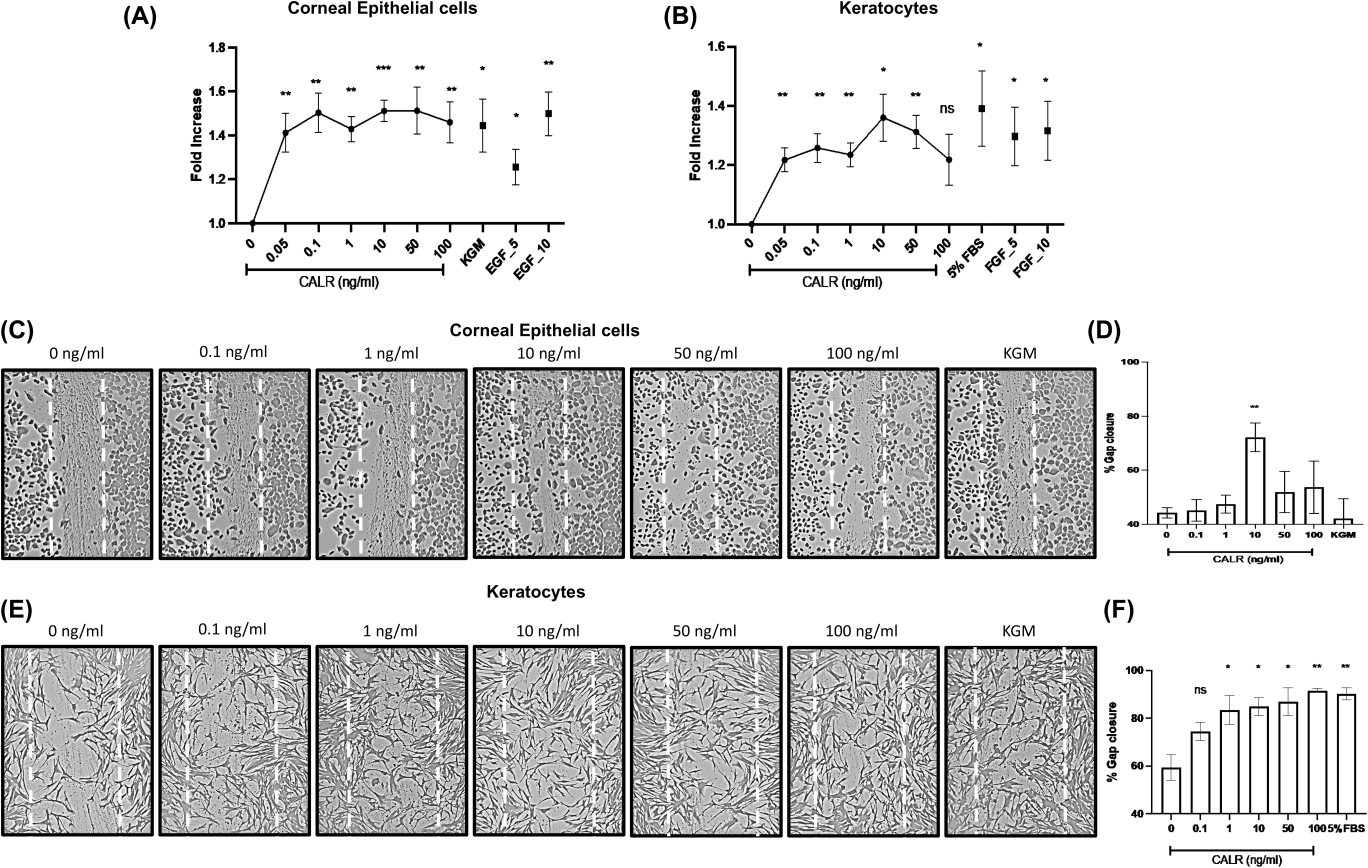
Calreticulin stimulates proliferation and migration of corneal epithelial cells and stromal keratocytes in vitro. (A, C, D) Corneal epithelial cells (CE) and (B, E, F) stromal keratocytes. Proliferation: CE cells and keratocytes were synchronized for 24 h in basal media in 96‐well plates in triplicate and then, treated with increasing concentrations of CALR (0–100 ng/mL) in keratinocyte basal medium (KBM) or for keratocytes in DMEM‐F12 containing 0.5% FBS respectively, for 24 h. *Proliferation*: (A) CE cells: Controls: KBM‐2 (negative), epidermal growth factor (EGF) at 5 and 10 ng/mL (positive). (B) keratocytes: Controls: DMEM‐F12 (negative) and DMEM‐F12 containing 5%FBS or FGF (positive). CCK‐8 solution was added and cells incubated for 2 h; absorbance was measured at 450 nm. Cellular proliferation was quantified as fold increase relative to the negative control set at 1.0. CALR stimulates proliferation with peak activities for CE cells at 50 pg/mL and keratocytes at 10 ng/mL. *Migration*: In vitro wound healing scratch plate assay: cell monolayers in 24‐well plates were wounded with a 200 μL pipette, detached cells removed by PBS wash, followed by treatment with increasing concentrations of CALR, as described.^6^ Mitomycin C (5 μg/mL; 1 h) was used in initial experiments to ensure that migration did not involve proliferation. (C, D) CE cells: controls: KBM‐2 basal media (negative) and KGM (positive). Cells were incubated for 8 h. (E, F) Keratocytes: controls: DMEM‐F12 with 5% FBS (positive). Cells incubated for 16 h. (C–F). For measuring migration, the cells were stained with Coomassie blue in 10% acetic acid, 45% methanol for 15 min, washed with PBS, and grey scale images captured using Olympus CK2 microscope. Wound area was determined using ImageJ macros wound healing tool. Per cent wound closure was calculated for each treatment group by measuring the area of the wound (pixels) at 0 time compared to the end of the experiment. Statistical analysis was performed using the unpaired students *t*‐test. Data are represented as Mean ± SEM of three independent experiments. CALR stimulates migration of CE cells and keratocytes at peak concentrations of 10 and 1.0 ng/mL, respectively. The difference in epithelial cuboidal and stromal spindle morphology between the CE cells in Figure 5C and keratocytes in Figure 5E is well‐displayed. Data is represented as Mean ± SEM; *n* = 3. [*p* ≤ 0.05 (*); *p* ≤ 0.01 (**); *p* ≤ 0.001 (***); *p* ≤ 0.0001 (****)].

Essential mechanisms to the wound healing process are the migration of epithelial cells to resurface the open wound and fibroblasts into the wound to produce matrix proteins for reconstruction/regeneration of the wound defect and, cellular proliferation to increase the number of functional cells. As shown in Figure 4A, compared to the KBM control, CALR at 0.05 ng/mL for 24 h stimulated proliferation of synchronized CE cells by 1.5‐fold, which was maintained as a threshold until the final dose of 50 ng/mL (*p* ≤ 0.01). The extent of stimulation of proliferation was similar to the positive controls of 10 ng/mL EGF and complete KGM containing growth factors. CE cells were unresponsive to lower concentrations of CALR (below 0.05 ng/mL to 0.01 ng/mL; not shown). Additionally, CALR dose‐dependently stimulated proliferation of primary CE cells with a peak at 0.01 ng/mL (Figure S1). Figure 4B shows that compared to the negative control of media containing 0.5% FBS, CALR stimulated proliferation of keratocytes with a peak response of 1.4‐fold at 10 ng/mL (*p* ≤ 0.05), which dose‐dependently decreased at higher concentrations of CALR and the extent of proliferation was similar to the positive controls, 5%FBS and FGF at 5 and 10 ng/mL.

Using the standard wound healing scratch plate assay, as shown in Figure 4C and the graph in Figure 4D, CALR, after 8 h, induced migration of CE cells with a statistically significant single point response at 10 ng/mL (*p* ≤ 0.01), representing 72% closure of the wound compared to 44% in the KBM negative control. Thus, CALR was 1.6‐fold more potent in inducing migration than both the positive (KGM) and negative (KBM) controls. (72% vs. 44%). The keratocytes migratory response to CALR shows 83.6% closure of the wound gap at 1 ng/mL at 16 h incubation compared to 59.4% for the media control (0.5% FBS; *p* ≤ 0.05); a threshold response was maintained until 100 ng/mL (*p* ≤ 0.01; Figure 4E,F). The CALR response was 1.4‐fold greater than the negative control and the equal to the positive control, 5% FBS. Therefore, based on the multitude of growth factors in serum, CALR, in vitro, as a single protein, had similar potency as a putative maximum migratory effect on keratocytes.

### 3.5 Calreticulin accelerates corneal healing without fibrogenesis: fibrogenic protein markers in response to CALR in vitro parallel in vivo scoring and histology

**FIGURE 5:**
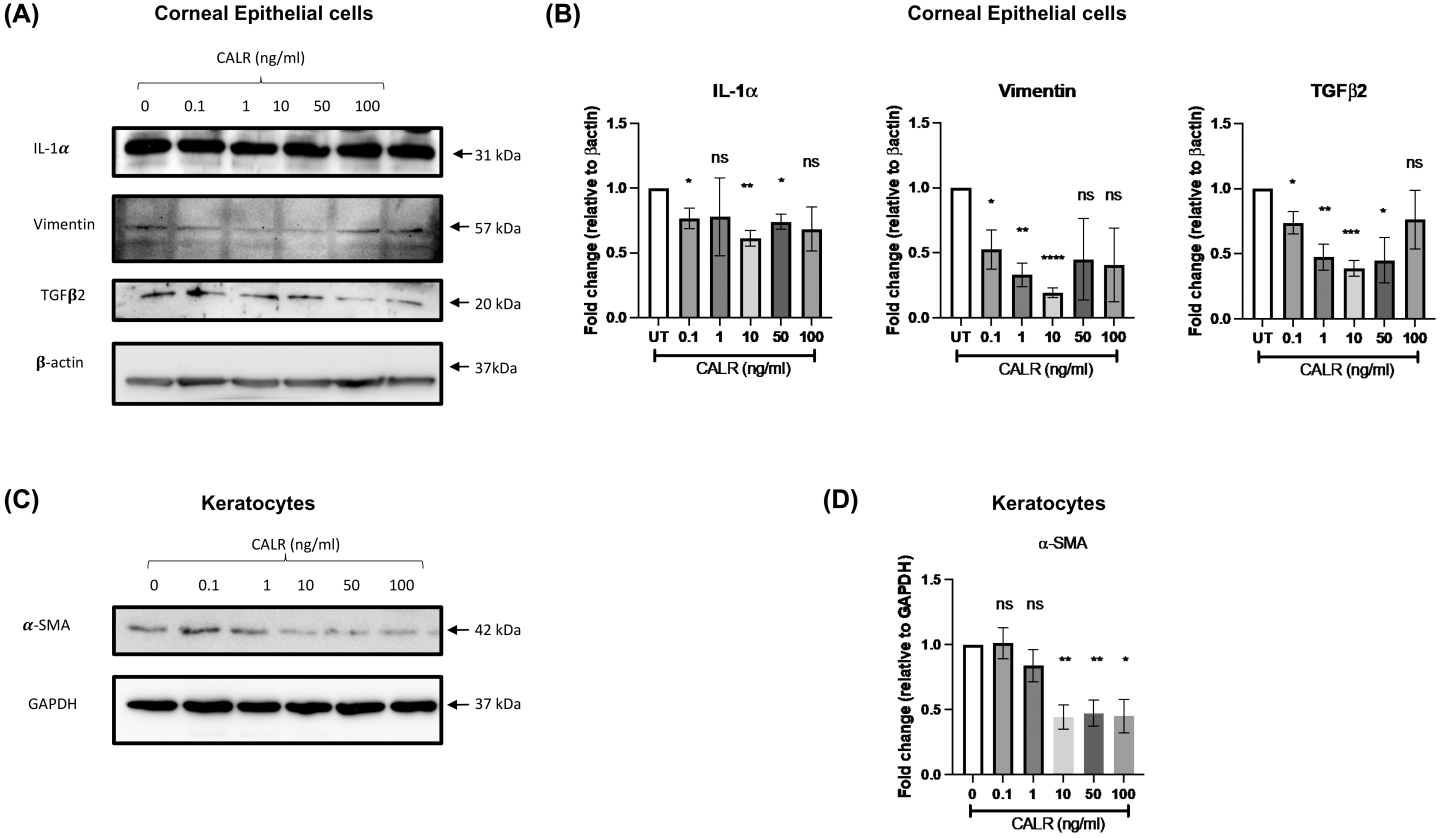
Exogenous Calreticulin decreases critical markers for fibrosis, vimentin and TGF‐β2 in corneal epithelial cells and α‐smooth muscle actin in keratocytes in vitro, by immunoblot analysis. (A, B) Corneal epithelial cells and (C, D) corneal stromal keratocytes cultured in KBM and DMEM‐F12 containing 0.5% FBS, respectively, at 70%–80% confluency in 6‐well plates, were treated with increasing concentrations of CALR (0–100 ng/mL) for 24 h, and cell lysates prepared on ice with 1X RIPA buffer (#20188, SigmaMillipore) containing undiluted protease inhibitor cocktail (Sigma, #P8340). (A, C) Protein concentrations were determined by the Micro‐BCA protein assay kit (Pierce) and 15 μg/well in Laemmli buffer containing 5% β‐mercaptoethanol were electrophoresed by SDS‐PAGE (10% acrylamide), and then, transferred to a polyvinylidene fluoride (PVDF) membrane for immunoblotting. Membranes were blocked with 5% nonfat dry milk in tris‐buffered saline (TBS) with 0.1% Tween‐20 (blocking buffer; TBST) for 1 h followed by overnight incubation in primary antibodies at 4°C. The following primary antibodies diluted in 5% milk were used: Mouse vimentin (#sc6260, Santa Cruz Biotechnology) at 1:1000 or mouse anti‐α‐smooth muscle actin (α‐SMA; #A5228, Sigma‐Aldrich). For rabbit anti‐human TGF‐β2 peptide antibody, the membrane was blocked overnight in 5% nonfat dry milk at 4°C followed by incubation with the antibody at 5 μg/mL in TBST containing 3% of nonfat milk overnight at 4°C, as described.^56^ As loading controls for all immunoblots, anti‐β‐actin at 1:10,000 or anti‐GAPDH (#sc‐32,233, Santa Cruz Biotechnology) at 1:2000 in 5% nonfat dry milk/TBST were used blots for CE cell and keratocytes, respectively. After incubation with primary antibodies, the membranes were probed with the appropriate secondary antibody for 1.5 h; either goat anti‐mouse IgG (Invitrogen), 1:2000 in TBST in 5% nonfat dry milk or goat anti‐rabbit IgG (Invitrogen), 1:2000 in TBST in 5% nonfat dry milk. Protein detection was performed using chemiluminescence‐based SuperSignal West Femto Maximum Sensitivity Substrate (#34095, Invitrogen ThermoScientific) (B) CE cells (TGF‐β2, vimentin) **(**D) Keratocytes (α‐SMA): Images of blots were captured using a ChemiDoc MP Imaging System (Bio‐Rad) and protein levels for each sample in each well on the blots were determined by densitometry using ImageJ. Target protein band intensity was normalized compared to the intensity of β‐actin or GAPDH. Data is expressed as fold (y axis) change for each target protein with the untreated control assigned as one. Data is represented as Mean ± SEM; *n* = 3.

**FIGURE 6:**
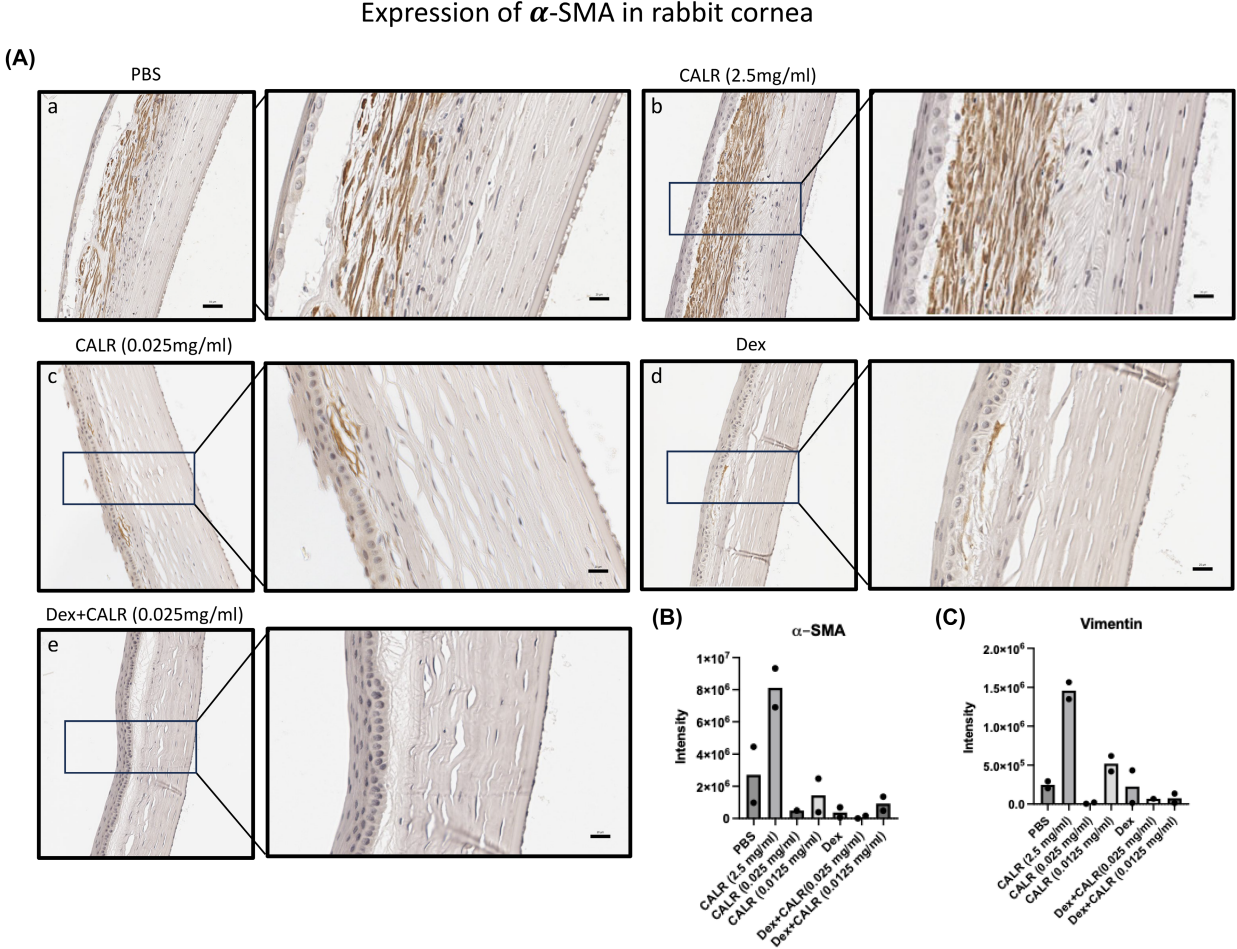
Calreticulin prevents the induction of fibrotic protein 𝜶‐SMA in vivo. (A) Paraffin embedded tissue sections 5.0 μm thick were placed on slides (NYU Experimental Pathology Core), sections baked overnight at 55°C, processed from xylene through graded alcohol and rehydrated. Endogenous peroxidase activity was quenched with 0.6% hydrogen peroxide for 30 min at RT followed by blocking non‐specific antigens with 3% normal goat serum in TBS containing 0.5% BSA (blocking buffer) for 20 min at RT. Primary antibodies were diluted in blocking buffer as follows: anti‐α‐SMA, 1:150, (#A5228, Sigma) and anti‐vimentin, 1:150 (#sc6260; Santa Cruz Biotechnology). The slides were incubated overnight with primary antibodies at 4°C, followed by goat‐anti‐mouse secondary antibody at 1:200 dilution for 1 h at RT, and protein expression levels detected using the VECTASTAIN® Elite® ABC‐HRP Kit; (Vector Laboratories) and DAB substrate (ab64238, Invitrogen, Abcam). Tissue sections were counterstained with haematoxylin (Modified Mayers; #ab220365, Abcam). Following graded dehydration to xylene, the slides were mounted with Permount Mounting Media (#SP15‐500, Fisher Chemical™ Co), imaged using Zeiss Axiophoton II microscope. Images of cornea at 20× and 50× digital magnification are shown for expression of 𝜶‐SMA in the rabbit eyes treated with PBS (Panel a); CALR (2.5 mg/mL; Panel b) CALR (0.025 mg/mL; Panel c); Dexamethasone (0.1%); (Panel d); Dexamethasone (0.1%) plus CALR (0.025 mg/mL); (Panel e). (B) Quantification of 𝜶SMA and (C) vimentin expression levels in rabbit corneal tissue sections. The intensity of protein expression was quantified by measuring intensity of DAB staining in each tissue section for each treatment group shown, by ImageJ. Data represents the intensity of region(s) of interest within each tissue section denoted by solid circles within each bar on the graph.

The responses of CE cells and keratocytes to CALR in vitro, with respect to expression of proteins that reflect corneal injury were determined by immunoblotting (Figure 5A). The densitometry of the immunoblots in Figure 5B shows that at the peak response of 10 ng/mL, CALR elicited a 50% and 75% decrease in the levels of the profibrogenic proteins, TGF‐β2 (*p* ≤ 0.001) and vimentin (*p* ≤ 0.0001). Figure 5C shows that CALR dose‐dependently decreased the level of the myofibroblast marker, α‐SMA in keratocytes. Taken together, CALR treatment of CE cells reduces TGFβ2 and in addition, directly decreases α‐SMA expression in keratocytes. Immunostaining for α‐SMA of the rabbit eyes treated with CALR and PBS, as the vehicle control representing base normal healing, and Dex controls, as treatment for post‐surgical inflammation are shown in Figure 6A. Consistent with Figure 4 (corneal fibrosis scores) showing that the treatment of rabbit corneas with the high concentration of CALR of 2.5 mg/mL is associated with a fibrotic response, Figure 6, Panel b illustrates a large number of keratocytes intensely staining for α‐SMA in the centers of the cornea treated with 2.5 mg/mL CALR. Comparably, by intensity of staining, Figure 6B, 0.025 mg/mL CALR (Panel c) and Dex (Panel d) show 3–10 and 1–4 times less intensity of immunostaining for α‐SMA and vimentin, respectively, than the PBS buffer‐treated control (Panel a). The level of expression of α‐SMA [and vimentin] in the corneal tissues varies directly with assessment of fibrosis from both the OCT scores for fibrosis (Figure 3C,D) and the histological analyses of the stained slides (Table 1) of corneas treated with the high concentration of CALR at 2.5 mg/mL. Notably, in agreement with these scores for fibrosis, 0.025 mg/mL CALR plus Dex‐treated corneal tissues (Panel e) lack the presence of α‐SMA immunostaining of keratocytes in the stroma as compared to the Dex alone treated rabbit corneas (Panel d).

## 4 DISCUSSION

The rabbit corneal injury model used in this study suffices for any injury or surgical procedure involving the epithelium through to the EBM (Bowman's membrane) and partial injury into the stroma such as, the damage incurred by PRK, including Lasik surgery, commonly used for vision correction.^9^, ^23^ This is the first study to show that topical treatment with CALR improves the rate of healing of corneal injury eliciting healing responses from both the epithelial and stromal cells of the rabbit cornea. CALR accelerates closure of rabbit corneal wounds 3 days faster than the Tris buffer‐treated control and 6 days faster than the steroid, Dexamethasone (Dex), used as SOC to mitigate inflammation that can lead to subsequent corneal fibrosis called hazing of the eye.^9^ Epithelial cell renewal of the cornea involves the ascendance and differentiation of cells from the basal layers of the epithelia, as occurs in cutaneous healing and in addition, stem cells from the limbus (Figure 1A) differentiate during migration to the central cornea.^24^ The production and remodelling of specific collagens, types I and V is crucial for healing of the stroma as the fibres are organized specifically to maintain essential transparency.^10^, ^25^ Therefore, the data suggest that CALR‐ treatment conformed to the specific requirements for healing of the cornea. In vitro, human CE cells and stromal keratocytes were shown to migrate, proliferate and synthesize fibronectin (data not shown) in response to CALR, thereby mechanistically supporting the acceleration of the corneal wound healing response to topical CALR obtained in vivo.

Inflammation, as part of the normal healing process can become dysregulated leading to hazing.^9^, ^10^ As such, the extent of inflammation and fibrosis following administration of potential therapeutics for injury to the eye, is a standard evaluation. Whereas the cornea is considered to have both immune and angiogenic privilege compared to the healing of other tissues due to the potential for compromising vision, this barrier can break down during severe inflammation.^9^ In the determination of dosing and efficacy of CALR in healing of corneal injury with CALR doses ranging three logs from 2.5, 0.25 and 0.025 mg/mL, only the highest dose caused ocular surface irregularities, inflammatory infiltration and stromal fibrotic changes (Table 1). However, CALR treatment with a 10‐fold difference in concentration (0.25 and 0.025 mg/mL) both, elicited accelerated wound closure at an average of 91 h post‐wounding compared to 142 h for the buffer control with mild inflammation and fibrosis scores similar to the control. Therefore, CALR at 0.25 mg/mL (8750 ng), twice daily for 14 days for a total of 245 μg compared to 10 times less CALR at 0.025 mg/mL (875 ngs) elicited the same magnitude of biological activity in wound closure without causing fibrosis. As the CALR‐treated rabbit corneas healed with only mild inflammation and fibrosis except at the highest dose of 2.5 mg/mL (total 2.45 mgs), inductive fibrotic effects of topical CALR to the cornea do not appear to be a concern for CALR as a biotherapeutic to accelerate corneal healing. However, a dosing regimen still needs to be established.

In contrast to exogenous CALR seemingly anti‐fibrotic effects in tissue regeneration,^6^ intracellular ER CALR has been shown to be involved in fibrosis in different organ systems due to its key role in mediating TGF‐β‐induced ECM induction, including atherosclerotic lesions and the foreign body response, with TGF‐β‐mediated signalling requiring ER‐mediated calcium release and NFAT activation.^26^, ^27^, ^28^, ^29^ These experiments used CALR null mouse embryo fibroblasts that lacked the TGF‐β‐induced fibrotic response in contrast to the wild type MEFs in which TGF‐β increased levels of collagen 1 and fibronectin. By contrast, topically applied CALR accelerates corneal ophthalmic repair of injury with full wound closure without causing fibrosis at the two lower concentrations used herein. Similarly, excisional full thickness mouse wounds healed by a tissue regenerative process with lack of scarring following topical application of CALR^6^ and in vitro, exogenous CALR attenuated TGF‐β‐induction of ECM proteins by fibroblasts.^20^ The role of CALR in fibrosis is complex as in early classic experiments using inductive models of organ‐specific fibrosis, CALR was shown to be upregulated in animal fibrosis models of rat renal tubules and lung but not in a cardiac model.^30^ In addition, TGF‐β upregulated CALR in vitro in human proximal tubule cells and more recently, mechanisms regulating CALR transcription were elucidated in renal fibrosis.^31^ Therefore, opposite roles of CALR in skin and eye modulation of fibrosis versus lung and kidney induction of fibrosis implicates organ‐specific effects. Understanding mechanisms involved in both the agonistic or antagonistic targeted effects of CALR on fibrosis present important therapeutic avenues to pursue.

Interestingly, the in vitro cellular functional responses to exogenous CALR are mediated by an outside‐in receptor signalling mechanism requiring intracellular ER‐CALR.^20^ Signalling mechanisms for exogenous CALR varied functions is largely unknown. However, an endocytic mechanism involving TSP‐1, CALR and LRP‐1 interaction enabling LRP‐1 signalling via membrane microdomain lipid rafts was elegantly shown by molecular dynamic simulations.^32^, ^33^ Whether TSP‐1 or the TSP‐1‐CALR‐LRP‐1 co‐receptor complex, or CALR‐LRP1 is involved in corneal wound healing in vivo or in the in the vitro activities shown here was not investigated. In a separate study, we have shown that LRP1 signalling is involved in ECM, TGF‐β1 and TGF‐β3 induction by CALR in dermal fibroblasts.^20^ As demonstrated for CE cells and stromal cells here, CALR has been shown to stimulate proliferation and migration of human and mouse cutaneous keratinocytes and dermal fibroblasts.^5^, ^6^

The use of both steroids and antibiotics for ocular procedures or injuries is clinically routine.^22^ However, an adverse effect of corticosteroids is delayed healing, which increases the probability of infection, suppresses the immune response, prolongs pain and can cause temporary visual loss.^34^ CALR (0.025 mg/mL) increased wound closure in combination with Dex compared to Dex alone showing a trend. The last animal to heal in the Dex plus 0.025 mg/mL CALR‐treated eyes was 5 days sooner than the Dex alone‐treated group. The variation in wound size over time of individual animals and the lack of obtaining data for time to complete closure for all animals affected estimating statistical significance in terms of differences in rate of closure in the Dex combination experiments; the CALR 0.025 plus Dex healed on an average of 1.7 days sooner than Dex alone. Notably, Dex was applied for 7 days (BID) and CALR (QD) for 4 days, which might have influenced the ability of CALR to statistically improve the Dex‐related delay in healing. Further studies, with higher or more prolonged dosing with CALR are necessary to pursue in combination with Dex. Interestingly, CALR plus Dex reduced the inflammatory infiltrate, corneal thickening, corneal disorganization and fibrosis caused by the injury better than Dex alone [and PBS control] suggesting that CALR by itself might have anti‐inflammatory or anti‐fibrotic effects on corneal injury, which is exciting to further interrogate. The use of CALR as a potential therapeutic in combination with Dex has extensive application. For example, when the immunosuppressive effect of DEX is a problem such as, with herpetic lesions of the eye, CALR could both accelerate healing and decrease inflammation and fibrosis in the absence of Dex. CALR mediates membrane ruffling and phagocytosis by macrophages^35^, ^36^, ^37^ and is an opsonin for bacteria.^38^ Therefore, infectious keratitis ulcerations, a prominent ophthalmic pathology, which is the 5th leading cause of blindness, caused by contact lenses and injuries, should benefit from the antimicrobial effects of CALR.^39^, ^40^, ^41^ Other ophthalmic therapeutic applications of CALR for repair of corneal injury include CE defects, all types of PRK, corneal transplants, corneal abrasions from dry‐eye, post‐surgery involving the ocular surface and systemic disease affecting the eye such as diabetic keratopathy.^42^, ^43^, ^44^ As extensive data on the CALR fragment (amino acids 135–164) termed vasostatin and also, intact CALR, show inhibition of angiogenesis via suppression of endothelial cell proliferation and VEGF levels, the action of CALR in the posterior portion of the eye for the treatment of macular degeneration, currently treated with anti‐VEGF Mabs, which have complications, should be investigated.^45^, ^46^, ^47^ For support of this use, gene delivery of the vasostatin domain inhibited ocular neovascularization.^48^

The numbers of myofibroblasts in a healing corneal wound is commensurate with the extent of fibrosis.^8^, ^9^, ^15^ Accordingly, and consistent with the highest inflammatory response and fibrosis, the corneal stroma of the 2.5 mg/mL CALR‐treated rabbit eyes (Exp #1, BID for 14 days) showed numerous myofibroblasts expressing α‐SMA (Figure 6). In contrast, CALR with Dex showed notably reduced numbers of α‐SMA expressing cells in the rabbit corneas. Interestingly, the PBS control had the greatest number of α‐SMA positive cells in Exp#2 whereas CALR 0.025 mg/mL and Dex‐treated eyes had little to no positive stromal cells, which was less than Dex alone. The in vivo results were mechanistically supported in vitro by showing that CALR decreased the levels of TGF‐β2 by human CE cells and the levels of α‐SMA by keratocytes in 24 h cultures (Figure 5). Thus, in vivo, CALR treatment might decrease TGF‐β2 for a lesser effect on the conversion of keratocytes to myofibroblasts [associated with fibrosis] and in addition, block α‐SMA expression by keratocytes suggesting a double effect on decreasing the fibrotic response. Importantly, the amount of TGF‐β2, the number of cells that convert to myofibroblasts, and the duration of the presence of these cells during the wound healing response, control the severity of the fibrotic response and notably, myofibroblasts undergo apoptosis in the absence of TGF‐β2.^8^, ^49^ Therefore, the in vitro results support the general lack of fibrosis/scarring with topical CALR treatment of the cornea.

Nanofibers (NFs) composed of various polymers that are electrospun such as, blended hyaluronic acid/PVA inserts or bioactive self‐assembled NFs, for example, composed of laminin and fibronectin have been successfully used to prolong drug delivery for ophthalmic bioengineering of therapeutics.^50^, ^51^, ^52^, ^53^ NFs, nanoparticles, and chemical implants^52^ can be engineered to be transparent, have optimal local release characteristics, and incorporate bioactive therapeutic agents of diverse functions including antibiotics and steroids.^54^ Recently, we electrospun CALR into PCL/Collagen 1 NFs with full retention of cutaneous wound healing‐related biological activities by human dermal keratinocytes and fibroblasts.^55^ Therefore, with CALR functional and structural stability and modifiable release kinetics following electrospinning into NFs, this option for CALR drug development for corneal surface wounds has precedence and should be explored. The combined effect of CALR on the eye in accelerating wound healing and preventing fibrosis underscores this protein's potential use as a biotherapeutic for numerous ophthalmic pathologies.

## AUTHOR CONTRIBUTIONS

**Sarita Mishra:** Data curation (equal); formal analysis (equal); investigation (equal); methodology (equal); validation (equal); visualization (equal); writing – review and editing (equal). **Miguel A. Manzanares:** Data curation (equal); formal analysis (equal); investigation (equal); methodology (equal); validation (equal); visualization (equal); writing – review and editing (equal). **Justin Prater:** Data curation (equal); formal analysis (equal); investigation (equal); methodology (equal); project administration (supporting); resources (supporting); supervision (equal); validation (equal); visualization (equal); writing – review and editing (equal). **David Culp:** Data curation (equal); formal analysis (equal); investigation (equal); methodology (equal); project administration (equal); resources (supporting); supervision (equal); validation (equal); visualization (equal); writing – review and editing (equal). **Leslie I. Gold:** Conceptualization (lead); data curation (equal); formal analysis (lead); funding acquisition (lead); investigation (lead); methodology (equal); project administration (lead); resources (lead); supervision (lead); validation (lead); visualization (lead); writing – original draft (lead); writing – review and editing (lead).

## FUNDING INFORMATION

Tissue Regeneration Sciences, Inc.

## CONFLICT OF INTEREST STATEMENT

The authors declare no competing interests.

## Supporting information


**Data S1.** Supporting Information.

## Data Availability

The datasets used and/or analyzed for this study submitted for publication are available from the corresponding author on reasonable request.
