# Supplementary material for: Calreticulin accelerates corneal wound closure and mitigates fibrosis: Potential therapeutic applications
Source: J Cell Mol Med. 2023 Nov 20;28(5):e18027. doi: 10.1111/jcmm.18027 (PMC10902309; doi:10.1111/jcmm.18027)
Supplement: Supplementary file 1 — Data S1. Supporting Information. [file JCMM-28-e18027-s001.pptx]

## Slide 1
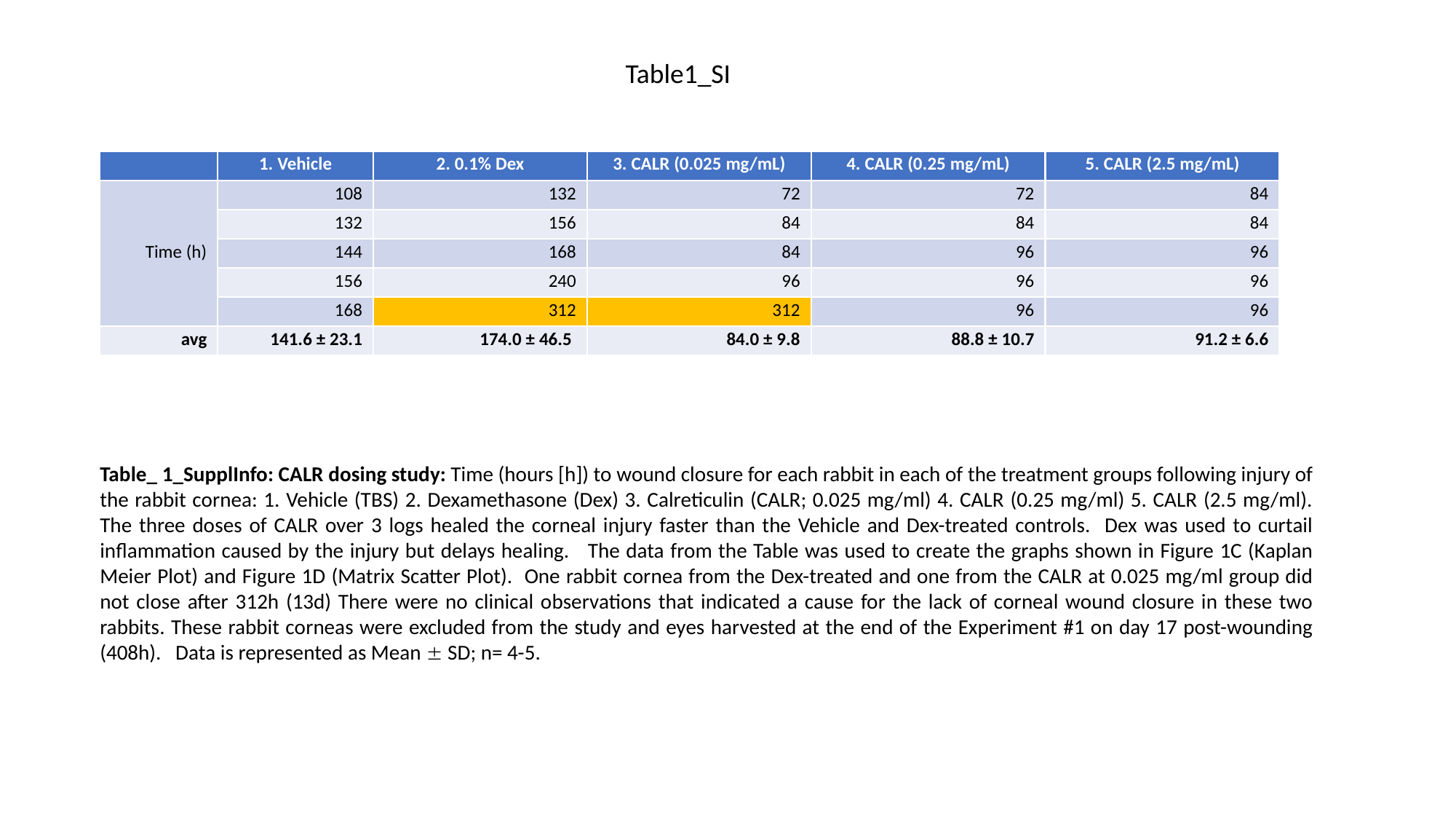

Table1_SI
| ​ | 1. Vehicle​ | 2. 0.1% Dex​ | 3. CALR (0.025 mg/mL)​ | 4. CALR (0.25 mg/mL)​ | 5. CALR (2.5 mg/mL)​ |
| --- | --- | --- | --- | --- | --- |
| Time (h)​ | 108​ | 132​ | 72​ | 72​ | 84​ |
| | 132​ | 156​ | 84​ | 84​ | 84​ |
| | 144​ | 168​ | 84​ | 96​ | 96​ |
| | 156​ | 240​ | 96​ | 96​ | 96​ |
| | 168​ | 312​ | 312​ | 96​ | 96​ |
| avg​ | 141.6 ± 23.1​ | 174.0 ± 46.5 ​ | 84.0 ± 9.8​ | 88.8 ± 10.7​ | 91.2 ± 6.6​ |
Table_ 1_SupplInfo: CALR dosing study: Time (hours [h]) to wound closure for each rabbit in each of the treatment groups following injury of the rabbit cornea: 1. Vehicle (TBS) 2. Dexamethasone (Dex) 3. Calreticulin (CALR; 0.025 mg/ml) 4. CALR (0.25 mg/ml) 5. CALR (2.5 mg/ml). The three doses of CALR over 3 logs healed the corneal injury faster than the Vehicle and Dex-treated controls. Dex was used to curtail inflammation caused by the injury but delays healing. The data from the Table was used to create the graphs shown in Figure 1C (Kaplan Meier Plot) and Figure 1D (Matrix Scatter Plot). One rabbit cornea from the Dex-treated and one from the CALR at 0.025 mg/ml group did not close after 312h (13d) There were no clinical observations that indicated a cause for the lack of corneal wound closure in these two rabbits. These rabbit corneas were excluded from the study and eyes harvested at the end of the Experiment #1 on day 17 post-wounding (408h). Data is represented as Mean ± SD; n= 4-5.

## Slide 2
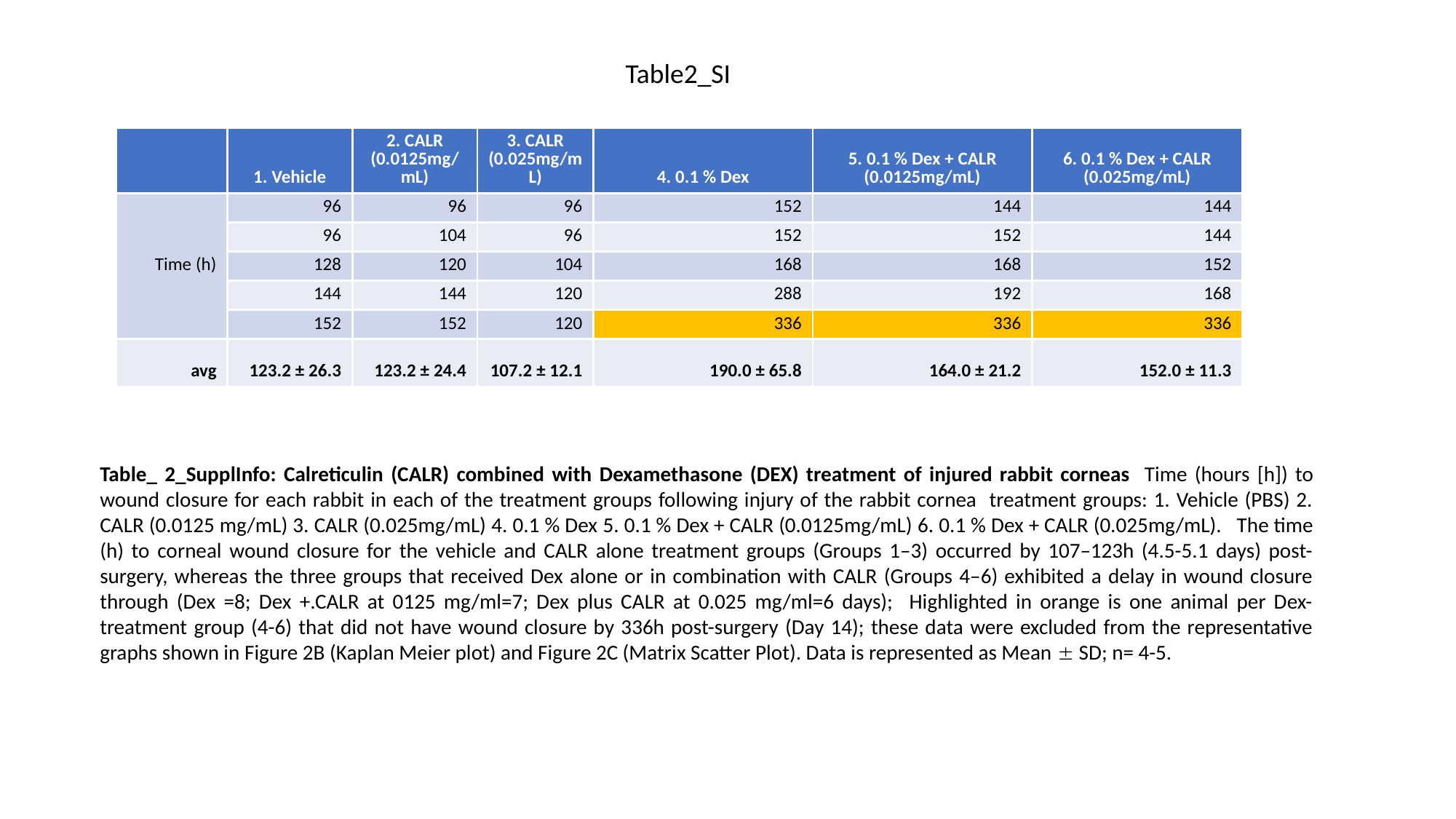

Table2_SI
| ​ | 1. Vehicle​ | 2. CALR (0.0125mg/mL)​ | 3. CALR (0.025mg/mL)​ | 4. 0.1 % Dex​ | 5. 0.1 % Dex + CALR (0.0125mg/mL)​ | 6. 0.1 % Dex + CALR (0.025mg/mL)​ |
| --- | --- | --- | --- | --- | --- | --- |
| Time (h)​ | 96​ | 96​ | 96​ | 152​ | 144​ | 144​ |
| | 96​ | 104​ | 96​ | 152​ | 152​ | 144​ |
| | 128​ | 120​ | 104​ | 168​ | 168​ | 152​ |
| | 144​ | 144​ | 120​ | 288​ | 192​ | 168​ |
| | 152​ | 152​ | 120​ | 336​ | 336​ | 336​ |
| ​ avg​ | 123.2 ± 26.3​ | 123.2 ± 24.4​ | 107.2 ± 12.1​ | 190.0 ± 65.8​ | 164.0 ± 21.2​ | 152.0 ± 11.3​ |
Table_ 2_SupplInfo: Calreticulin (CALR) combined with Dexamethasone (DEX) treatment of injured rabbit corneas Time (hours [h]) to wound closure for each rabbit in each of the treatment groups following injury of the rabbit cornea treatment groups: 1. Vehicle (PBS) 2. CALR (0.0125 mg/mL)​ 3. CALR (0.025mg/mL)​ 4. 0.1 % Dex​ 5. 0.1 % Dex + CALR (0.0125mg/mL)​ 6. 0.1 % Dex + CALR (0.025mg/mL). The time (h) to corneal wound closure for the vehicle and CALR alone treatment groups (Groups 1–3) occurred by 107–123h (4.5-5.1 days) post-surgery, whereas the three groups that received Dex alone or in combination with CALR (Groups 4–6) exhibited a delay in wound closure through (Dex =8; Dex +.CALR at 0125 mg/ml=7; Dex plus CALR at 0.025 mg/ml=6 days); Highlighted in orange is one animal per Dex-treatment group (4-6) that did not have wound closure by 336h post-surgery (Day 14); these data were excluded from the representative graphs shown in Figure 2B (Kaplan Meier plot) and Figure 2C (Matrix Scatter Plot). Data is represented as Mean ± SD; n= 4-5.

## Slide 3
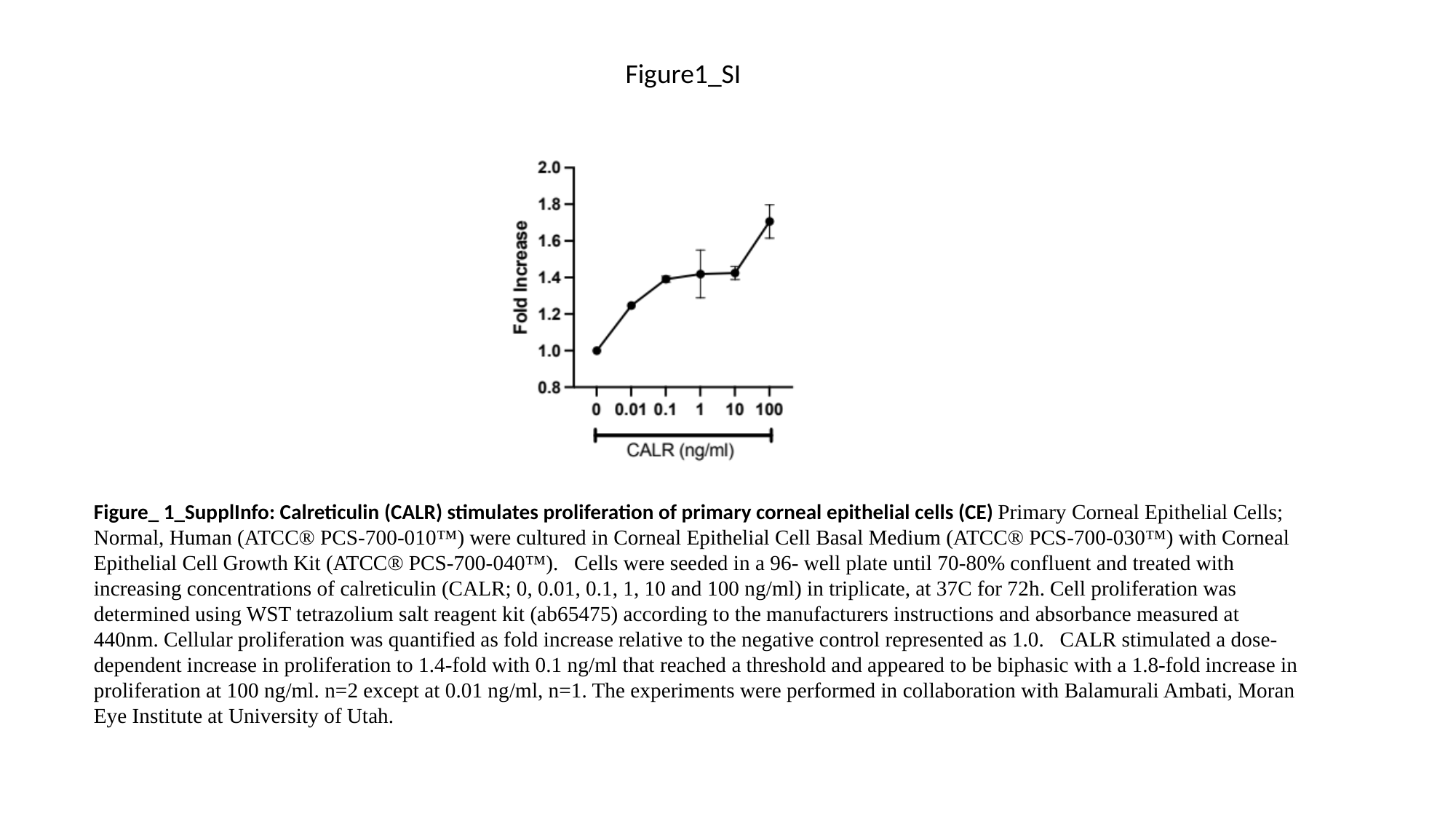

Figure1_SI
Figure_ 1_SupplInfo: Calreticulin (CALR) stimulates proliferation of primary corneal epithelial cells (CE) Primary Corneal Epithelial Cells; Normal, Human (ATCC® PCS-700-010™) were cultured in Corneal Epithelial Cell Basal Medium (ATCC® PCS-700-030™) with Corneal Epithelial Cell Growth Kit (ATCC® PCS-700-040™). Cells were seeded in a 96- well plate until 70-80% confluent and treated with increasing concentrations of calreticulin (CALR; 0, 0.01, 0.1, 1, 10 and 100 ng/ml) in triplicate, at 37C for 72h. Cell proliferation was determined using WST tetrazolium salt reagent kit (ab65475) according to the manufacturers instructions and absorbance measured at 440nm. Cellular proliferation was quantified as fold increase relative to the negative control represented as 1.0. CALR stimulated a dose-dependent increase in proliferation to 1.4-fold with 0.1 ng/ml that reached a threshold and appeared to be biphasic with a 1.8-fold increase in proliferation at 100 ng/ml. n=2 except at 0.01 ng/ml, n=1. The experiments were performed in collaboration with Balamurali Ambati, Moran Eye Institute at University of Utah.
